# Transitions Between Circulatory States After Out-of-Hospital Cardiac Arrest: Protocol for an Observational, Prospective Cohort Study

**DOI:** 10.2196/resprot.8558

**Published:** 2018-01-19

**Authors:** Halvor Langeland, Daniel Bergum, Magnus Løberg, Knut Bjørnstad, Jan Kristian Damås, Tom Eirik Mollnes, Nils-Kristian Skjærvold, Pål Klepstad

**Affiliations:** ^1^ Department of Anesthesiology and Intensive Care Medicine St. Olav’s University Hospital Trondheim Norway; ^2^ Department of Circulation and Medical Imaging Faculty of Medicine and Health Sciences The Norwegian University of Science and Technology Trondheim Norway; ^3^ Mid-Norway Sepsis Research Center Norwegian University of Science and Technology Trondheim Norway; ^4^ Institute of Health and Society, Department of Health Management and Health Economics Faculty of Medicine University of Oslo Oslo Norway; ^5^ KG Jebsen Center for Colorectal Cancer Research Department of Transplantation Medicine Oslo University Hospital Oslo Norway; ^6^ Clinic of Cardiology St. Olav’s University Hospital Trondheim Norway; ^7^ Centre of Molecular Inflammation Research Department of Clinical and Molecular Medicine Norwegian University of Science and Technology Trondheim Norway; ^8^ Department of Infectious Diseases St. Olav’s University Hospital Trondheim Norway; ^9^ KG Jebsen Inflammation Research Center Department of Immunology Oslo University Hospital Oslo Norway; ^10^ Research Laboratory Nordland Hospital Bodø Norway; ^11^ KG Jebsen Thrombosis Research and Expertise Center Faculty of Health Sciences University of Tromsø Tromsø Norway

**Keywords:** out-of-hospital cardiac arrest, critical care, hemodynamics, inflammation, biomarkers

## Abstract

**Background:**

The post cardiac arrest syndrome (PCAS) is responsible for the majority of in-hospital deaths following cardiac arrest (CA). The major elements of PCAS are anoxic brain injury and circulatory failure.

**Objective:**

This study aimed to investigate the clinical characteristics of circulatory failure and inflammatory responses after out-of-hospital cardiac arrest (OHCA) and to identify patterns of circulatory and inflammatory responses, which may predict circulatory deterioration in PCAS.

**Methods:**

This study is a single-center cohort study of 50 patients who receive intensive care after OHCA. The patients are followed for 5 days where detailed information from circulatory variables, including measurements by pulmonary artery catheters (PACs), is obtained in high resolution. Blood samples for inflammatory and endothelial biomarkers are taken at inclusion and thereafter daily. Every 10 min, the patients will be assessed and categorized in one of three circulatory categories. These categories are based on mean arterial pressure; heart rate; serum lactate concentrations; superior vena cava oxygen saturation; and need for fluid, vasoactive medications, and other interventions. We will analyze predictors of circulatory failure and their relation to inflammatory biomarkers.

**Results:**

Patient inclusion started in January 2016.

**Conclusions:**

This study will obtain advanced hemodynamic data with high resolution during the acute phase of PCAS and will analyze the details in circulatory state transitions related to circulatory failure. We aim to identify early predictors of circulatory deterioration and favorable outcome after CA.

**Trial Registration:**

ClinicalTrials.gov: NCT02648061; https://clinicaltrials.gov/ct2/show/NCT02648061 (Archived by WebCite at http://www.webcitation.org/6wVASuOla)

## Introduction

### Post Cardiac Arrest Syndrome

Recent advances in cardiopulmonary resuscitation (CPR) have improved the chance of successful return of spontaneous circulation (ROSC) after cardiac arrest (CA) [1,2]. This implies that patients, who previously died during CPR, now obtain ROSC and are admitted to hospitals for further treatment. However, in-hospital mortality is unchanged at around 50% to 70% [3-5]. The post cardiac arrest syndrome (PCAS) is responsible for the majority of in-hospital deaths after CA. PCAS includes (1) brain injury, (2) myocardial dysfunction, and (3) systemic ischemia and reperfusion injury. In addition, the pathological process that caused the CA and other chronic diseases influences the clinical course of PCAS. Circulatory support after CA usually follows recommendations similar to those used for treatment of septic shock [6-9]. Septic shock and PCAS share some characteristics and experiences from the treatment of septic shock, such as emphasis on oxygen deliverance, which may be useful [10,11]. However, PCAS is a different clinical entity than septic shock, and circulatory failure after CA may have special characteristics [4]. This is exemplified by the need to perfuse the postischemic brain without unnecessary strain on the postischemic heart.

### Inflammation and Circulatory Failure

Circulatory failure after CA is both due to cardiac etiologies and systemic inflammatory response elicited by the hypoxic insult and reperfusion, but the relative contribution of each factor is unknown. The balance between pro- and anti-inflammatory cytokine signals is important in the development of organ failure [12]. Several studies have explored cytokine plasma concentrations as biomarkers for severity, risk of organ failure, and mortality in sepsis [13-15]. By comparison, only 4 studies in humans, with data from 2 patient populations, have evaluated the inflammatory response in PCAS [10,16-18], and 2 studies have explored the endothelial activation and injury in relation to inflammation in PCAS [18,19]. Furthermore, none of these studies specifically investigated the relationship between endothelial injury, inflammatory response, and circulatory failure.

In a cohort study from 2002, 73 out of 165 normothermic patients treated for out-of-hospital cardiac arrest (OHCA) developed circulatory instability, with median onset 6.8 hours after OHCA. [20]. Low cardiac output (CO) and filling pressures characterized the circulatory instability. The CO improved after 24 hours but superimposed vasodilatation developed, requiring fluid administration and use of vasoactive medications. Most patients recovered within 3 days, and hemodynamic status did not predict neurologic outcome [20]. In a Norwegian study, these results were confirmed in hypothermic CA patients [21]. In contrast, in the *Target Temperature Management Trial*, which randomized CA patients to either 33°C or 36°C, the circulation of patients in both groups were characterized by vasoconstriction [5,22,23]. However, the variables in studies on circulatory failure after CA are highly dependent on the use of vasoactive medications, and some studies are difficult to interpret because of limited information about the use of these medications.

### Rationale for a New Study

The International Liaison Committee on Resuscitation consensus statement from 2008 acknowledges the lack of knowledge about optimal treatment of PCAS and how to best deliver circulatory support after CA [4].

This protocol describes a study aimed to investigate patients with ROSC after OHCA, regarding (1) characteristics of circulatory failure in PCAS; (2) the endothelial and inflammatory response in PCAS; and (3) the relationship between circulatory failure and the inflammatory and endothelial response in PCAS.

## Methods

### Research Questions

This study will address the following research questions:

- What are the characteristics of circulatory failure in PCAS?
- What are the characteristics of transitions between different clinical circulatory states during PCAS?
- What is the inflammatory response measured by inflammatory and endothelial biomarkers in PCAS?
- Which clinical characteristics and biomarkers predict changes in clinical circulatory states?

### Study Design

This study is a single-center, prospective, observational cohort study.

### Setting

The study will take place at the intensive care unit (ICU) and the coronary care unit (CCU) at the St. Olav’s University Hospital, a tertiary referral university hospital in Trondheim, Norway, with a catchment population of 700,000.

### Eligibility

#### Inclusion

All adult patients who are admitted to either the ICU or the CCU with obtained ROSC after OHCA will be considered for inclusion. Inclusion is performed immediately after arrival to the ICU or CCU.

#### Exclusion Criteria

Exclusion criteria are age <18 years, CA of septic or anaphylactic origin, sepsis within 24 hours before CA, pregnant women, transferred from other hospitals after OHCA, and decision to not initiate life-sustaining therapy after hospital arrival.

### Censoring

Patients are censored from further follow-up if the patient undergoes acute cardiothoracic surgery or intervention with extracorporeal membranous oxygenation support or a ventricular assist device, at the time of death, if life-prolonging therapy is withdrawn or withheld, or when the patient is transferred to a general ward or another hospital. The reason for censoring is recorded, and all data obtained until censoring will be included in the analysis.

### Sample Size

This is an observational study, and the frequency of abnormal biomarkers, circulatory states, and endpoints are largely unknown; therefore, no formal sample size calculation has been performed [24]. On the basis of a sample size from similar studies describing pathophysiology, we aim at including 50 patients [21,25].

### Routine Post Cardiac Arrest Syndrome Treatment

#### General Consideration

The treatment of the patients is decided by the physician in charge and will not be changed due to the participation in the study. The routine treatment after OHCA at St. Olav’s University Hospital will be applied unless there are specific indications for alternative strategies. Routine care is briefly outlined below.

##### Specific Cardiac Interventions

For OHCA suspected to be caused by disease in the coronary arteries, procedures for revascularization are routinely considered and performed if decided by the cardiologist. All use of medications for cardiac diseases and for anticoagulation is decided by the cardiologist in charge of treatment.

##### Therapeutic Hypothermia

According to the current practice at St. Olav’s University Hospital, the target temperature is usually 36°C. Active temperature management is performed for 24 hours.

##### Sedation and Analgesia

Sedation is initiated with either propofol or midazolam, and analgesia with either fentanyl or remifentanil. Sedation is titrated to Motor Activity Assessment Scale (MAAS) 0-1 during active temperature management and later titrated to the lowest dose achieving adequate patient comfort [26]. A muscle relaxant, cisatracurium, is not routinely used, but initiated if shivering during cooling or rewarming, and if needed to achieve adequate ventilation.

##### Cardiovascular Support

The primary treatment goal for circulatory support is to ensure adequate circulation, as evaluated by clinical examination (eg, tachycardia, pallor, cold skin, capillary refill), a mean arterial blood pressure (MAP) ≥65 mm Hg, and a urine output of ≥0.5 mL·kg^−1^·h^−1^. Generally, circulatory optimization is achieved through fluid and vasopressor administration after the following algorithm: in the presence of hypotension and/or tachycardia, the first step is to assess signs of tissue hypoperfusion (eg, cold, clammy skin and extremities, prolonged capillary refill time, diminished urine output, increasing lactate and decreasing base excess, decreasing central/mixed venous oxygenation, and if not sedated—deteriorating mental status). If the physician suspects tissue hypoperfusion, volume status is assessed through the presence of stroke volume variation >10% (pulsus paradoxus) and/or echocardiographic assessment of the heart and the inferior caval vein. If increased preload is indicated, repeated fluid boluses of 250 mL are given until cardiac output (CO) does not respond. If a fluid load is not indicated or does not improve the perfusion, vasoactive medication is administered. The standard vasoactive medications are norepinephrine and/or dobutamine, depending on whether vasoconstrictive and/or inotropic effects are indicated. If the vasoplegia is not improved by a high dose of norepinephrine (≥0.5 μg·kg^−1^·min^−1^), vasopressin (0.4 mU·kg^−1^·min^−1^) is considered.

##### Respiratory Support

Ventilation is administered by a SERVOi ventilator (Maquet Siemens, Germany). Ventilation is either pressure controlled or pressure supported for patients who have adequate spontaneous ventilation. The ventilator settings are adjusted to a positive end-expiratory pressure (PEEP) level, which give the best arterial oxygen partial pressure (PaO_2_)/fraction of inspired oxygen (FiO_2_) ratio (usually PEEP is set at 8 cm H_2_ O at the start of ventilation), a plateau pressure to give a tidal volume of 6-8 mL/kg, a respiratory rate to give a PaCO_2_ within the normal range, and a FiO_2_ to give a O_2_ saturation above 95%. Tracheostomy is considered for patients who are difficult to wean from mechanical respiratory support, usually on days 8-10 of the ICU or CCU stay.

##### Nutrition

No nutrition is given on the day of arrival to the ICU or CCU. Enteral nutrition is given as soon as possible in increasing doses (from 500 kcal/day to 1000 kcal/day to 2000 kcal/day). If enteral nutrition is not feasible or nutrition targets cannot be reached within approximately 4 to 6 days, total parenteral nutrition is gradually introduced on the fourth day (25 kcal·kg^−1^·day^−1^). Before nutrition is started, the patients receive glucose 100 g/daily. Metabolic control of blood sugar is aimed at 5-10 mmol/L by a continuous infusion of insulin. Patients who use insulin on a regular basis will receive insulin at all times, and hypoglycemia is corrected with increased glucose or nutrition administration.

##### Infection Control

Antibiotics are not given routinely, but they are introduced if there is a clinical suspicion of an infection.

##### Assessment of Hypoxic Cerebral Injury

Assessment of hypoxic cerebral injury is primarily based on the combination of clinical signs, serum neuron-specific enolase concentrations, and neurophysiological examination of somatosensory evoked potentials. Other examinations, for example, magnetic resonance imaging, are performed when required. The evaluation of potential hypoxic cerebral injury is usually initiated on the third or fourth day after CA.

##### Intensive Care Unit Procedures

Airway suction is routinely performed once daily to secure patent airway and as needed if there are signs of excessive airway secretions. Airway suction is performed with a 10, 12, or 14 French scale catheter (depending on endotracheal tube size) through a closed suction system.

Shift of position is routinely performed every third hour.

Spontaneous breathing trials (SBTs) are considered in patients who have spontaneous respiration, airway patency, reversal of the cause of respiratory failure, no uncontrolled infection or metabolic disturbance, heart rate <120, systolic BP 90-180 mm Hg, oxygen saturation >90%, FiO_2_ <0.5, and positive end-expiratory pressure (PEEP) ≤8 cmH_2_ O. The SBT is performed using pressure-supported ventilation with inspiratory pressure support of approximately 8 cmH_2_ O and PEEP 6-8 cmH_2_ O for 10-30 min.

### Study Procedure

#### Screening and Recruitment

**Figure 1:**
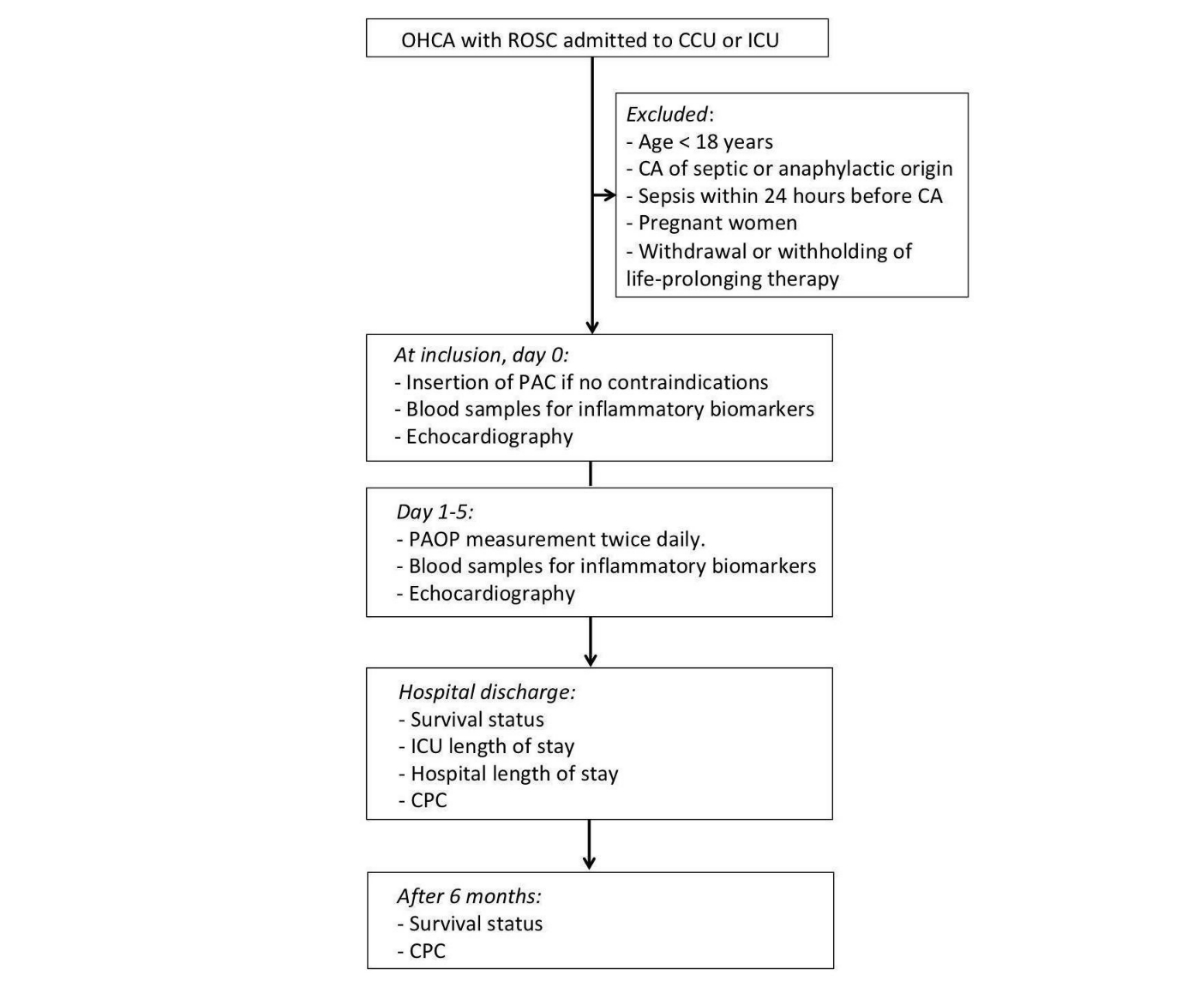
Flowchart summarizing patient enrollment and main study procedures. OHCA: out-of-hospital cardiac arrest; ROSC: return of spontaneous circulation; CCU: coronary care unit; ICU: intensive care unit; CA: cardiac arrest; PAC: pulmonary artery catheter; PAOP: pulmonary artery occlusion pressure; CPC: cerebral performance category.

Patients will be screened for eligibility and recruited at the time they are admitted to the ICU or CCU for intensive care after OHCA (Figure 1).

Demographic and clinical data will be extracted from the patient’s medical records and documentation from emergency medical service (EMS) personnel involved in the treatment of CA. Registrations of clinical variables, as part of routine critical care from the time of ICU/CCU arrival until inclusion, will be used.

If there is no contraindication to insert a pulmonary artery catheter (PAC), the catheter is inserted as early as possible after inclusion.

#### Baseline Variables

At time of inclusion the following variables will be registered:

- Patient characteristics: age, sex, ethnicity, weight, height, and premorbid cerebral performance category (CPC) [27,28]
- Charlson Comorbidity Index [29]
- Characteristics related to the CA (*Utstein Style Template*) [30]: location, witnessed arrest, time of emergency call, bystander CPR, time of EMS personnel arrival, initial monitored rhythm, time to first defibrillation, time to ROSC, presumed cardiac or noncardiac etiology, and medications given during or after CPR. End-tidal CO_2_ results, if applied
- Temperature at admission
- Known pulmonary aspiration during CPR
- Interventions

#### Registrations During the Study Period

Clinical data are registered in the electronic ICU chart, Picis Critical Care Manager (Optum Inc, USA). After inclusion, the following variables will be recorded:

At the time of inclusion and every minute:

- Basic vital measurements: heart rate and rhythm, invasive arterial blood pressure, pulmonary artery pressure, mixed venous oxygen saturation (SvO_2_, calibrated twice daily), central venous blood pressure, peripheral transcutaneous oxygen saturation, and temperature
- Central hemodynamic measurements: CO and systemic vascular resistance (SVR), and corresponding indexed values (related to body-surface area)
- Respiratory support: ventilator mode, respiratory rate, FiO_2_, minute ventilation, PEEP, and plateau pressure

At the time of inclusion and every following hour:

- Fluid balance: fluid administrations, transfusions, and urine output
- All medications

At the time of inclusion and every sixth hour:

- Arterial blood gas analysis, including electrolytes
- MAAS score

After 24 hours:

- Simplified acute physiology score (SAPS) II [31]

At the time of inclusion and twice daily:

- Pulmonary artery occlusion pressure
- Central venous and mixed venous blood gas

At the time of inclusion and daily:

- Standardized echocardiographic evaluation performed by a trained cardiologist
- Intra-abdominal pressure
- Clinical chemistry: white blood count, thrombocyte count, creatinine, blood urea nitrogen, C-reactive protein, troponin-T, probrain natriuretic peptide, bilirubin, albumin, and haptoglobin
- Blood samples for inflammatory and endothelial biomarkers
- Modified clinical pulmonary infection score (CPIS) [32]
- Sequential organ failure assessment (SOFA) score [33]
- Glasgow Coma Score [34]
- Percutaneous coronary intervention, dialysis, and/or aorta balloon pump
- Results from chest x-ray or other diagnostic imaging tools ordered during the hospital stay
- Other events during the study period (eg, arrhythmias and seizures)

#### Biomarkers

As part of the study, we will establish a biobank for analysis of inflammatory and endothelial biomarkers. Blood samples will be taken at inclusion and every morning the 5 following days. After gentle mixing, the blood samples are placed vertical for 30 min in ambient temperature, and then centrifuged at 2200 g for 10 min. The supernatants are stored at −80°C within 1 hour from the time of sampling. During the study period, additional full blood will be drawn and stored at −80°C.

We will analyze the following biomarkers: interleukin (IL)-1β, IL-1 receptor antagonist (IL1-ra), IL-2, IL-4, IL-5, IL-6, IL-7, IL-8, IL-9, IL-10, IL-12 (p70), IL-13, IL-15, IL-17, eotaxin, basic fibroblast growth factor, granulocyte-colony stimulating factor, granulocyte macrophage colony stimulating factor, interferon γ, interferon-inducible protein 10, monocyte chemotactic protein 1, macrophage inflammatory protein 1α and 1β, platelet derived growth factor-BB, regulated upon activation T cell expressed and secreted, tumor necrosis factor, vascular endothelial growth factor, syndecan-1, sE-selectin, heparan sulfate, hyaluronic acid, soluble trombomodulin, and sVE-cadherin. Other biomarkers of interest, identified later, may be included in the final analysis.

#### Follow-Up After Discharge From Hospital

Follow-up after discharge from the hospital will include the following:

- ICU length of stay.
- Hospital length of stay.
- Survival status at hospital discharge and after 6 months.
- CPC at hospital discharge and after 6 months.

### Ethical Considerations

Research on critically ill patients, who are sedated or comatose and, therefore, not able to provide consent, calls for special ethical considerations. For the study, it is vital that clinical information is obtained from the initial critical stage of the disease. This study will not increase the overall risk for the patients, and the study is justifiable according to the *World Medical Association’s Declaration of Helsinki* regarding *Ethical Principles for Medical Research Involving Human Subjects*, June 1964, and its later amendments [35]. We will include patients in the study when they are admitted to the ICU/CCU and at the earliest feasible moment ask for consent from their relatives. Patients, who regain competency to give an informed consent, will later be asked for a deferred consent. This procedure is approved by the Regional Committee for Medical and Health Research Ethics, Central-Norway Health Region (REK Midt, No. 2015/1807).

### Assessment of Safety

Besides establishing and calibrating PAC (Swan-Ganz CCOmbo, Edward Lifescience, USA) and drawing blood for analysis of inflammatory biomarkers at a maximum of 5 time points, the study does not involve other interventions differing from routine care after OHCA. The insertion of PAC induces some benefits and some risks. One of the benefits is the potential of improved circulatory monitoring and support, including a more precise administration of fluids and vasoactive medications. However, complications related to the placement of PAC have also been reported. In a Cochrane report, PAC was neither found to increase mortality or length of stay in the ICU or the hospital (high-quality evidence according to the Grading of Recommendations Assessment, Development and Evaluation (GRADE) system for both the findings). The Acute Decompensated Heart Failure Syndromes Registry compared 502 patients with decompensated heart failure and PAC with 502 controls [36] and observed that the risk of all-cause death was lower in the PAC group than the control group (hazard ratio 0.3, 95% confidence interval (CI) 0.13-0.7). In a large study from Leipzig where 3730 patients with PAC who underwent cardiac surgery, only 0.1% experienced serious complications [37], a similar rate as transesophageal echocardiography (0.2%) [38]. The nursing-staff at ICU and CCU are trained in PAC care and extra lectures in PAC use will be given before and during the study. Only doctors, competent of PAC use, will perform measurements of pulmonary artery wedge pressure. We believe that the patients included will experience neither benefit nor harm by participating in this study.

### Statistical Analysis Plan

#### Statistical Methods

Descriptive data are presented with either median or interquartile range, or mean with 95% CI, as appropriate. For missing observation of physiological variables (eg, blood pressure), a value corresponding to the mean of the observation before and after this time point will be applied. For biomarkers, the value 0 is attributed if a result is below the detection limits of the assay. Statistical analyses will be performed using Stata or R.

#### Circulatory State Transitions During Post Cardiac Arrest Syndrome

During critical illness, most patients go through different states of hemodynamic stability. We have categorized the circulatory state after CA in 3 different categories based on data obtained from routine care (Table 1). Every 10 min, a patient is classified according to the least favorable measurement at that time (eg, isolated MAP of 40 mm Hg is sufficient to classify a patient to have severely disturbed circulation).

We will validate the appropriateness of the defined categories against the cardiac index.

**Figure 2:**
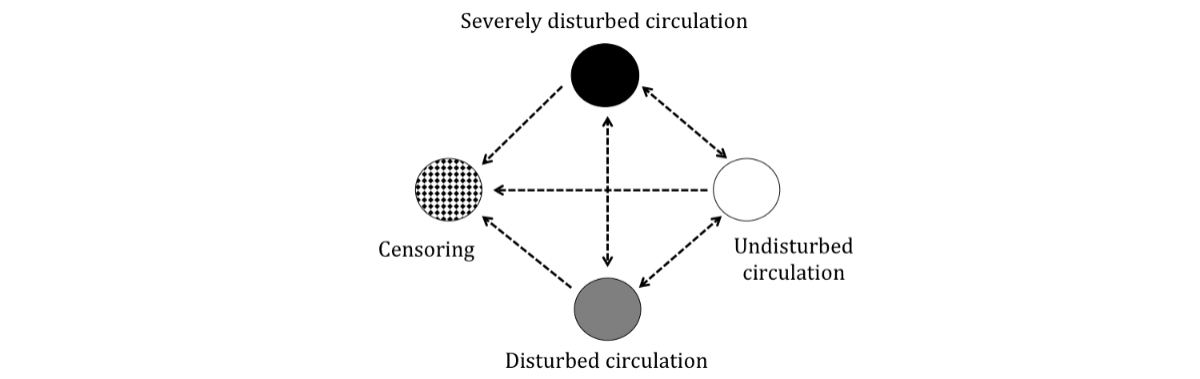
Possible transitions between circulatory states.

Patients may go back and forth between the clinical states, defined in Table 1, corresponding to the severity of their illness and its progression (Figure 2), and the changes from one clinical state to another will be recorded. By using survival analysis methodology, we allow for right censoring, which means that the information from patients with limited follow-up time can be included in the analyses. A patient who is in a certain clinical state at time *t* might remain in the same state or change to one of 2 others at time *t* +1. In such multi-endpoint settings, the clinical states compete with each other, and the transitions over time are best studied with methods developed for competing risk analyses.

**Table 1:** Circulatory states.

Variables	Circulation		
	Undisturbed	Disturbed	Severely disturbed
Mean arterial pressure, mm Hg	≥65	45-64	<45
Heart rate, bpm	51-100	<50, 101-130	≤40, >130
Lactate, mmol/L	<2	2–4	>4
ScvO_2_, %	≥65	50-64	<50
Fluid resuscitation, L/hours	<0.5	0.5-1.9	≥2
Norepinephrine, μg·kg^−1^·min^−1^	<0.1	0.1-0.29	≥0.3
Dobutamine, μg·kg^−1^·min^−1^	No	<10	≥10
Vasopressin	No	No	Yes
Epinephrine	No	No	Yes
Levosimedan	No	No	Yes
Aorta balloon pump	No	No	Yes

The Kaplan-Meier estimator accurately estimates the transition from one state to another, assuming that these 2 states describe all possible states. To be useful in a competing risk framework, the Kaplan-Meier estimator has been generalized into a matrix version, called the Aalen-Johansen estimator. To describe when, in time, most state transitions occur (ie, transition intensity), we will use the Nelson-Aalen estimator. The Nelson-Aalen estimator is a nonparametric estimator of the “cumulative hazard” of a given event, and it can be applied in a multi-state model.

#### Patterns Predictive of Deterioration in Circulatory State

We anticipate that not all patients will follow the same circulatory trajectory; during the study, some will deteriorate and some will improve in circulatory status, and during our observation, many will have several state transitions. We plan to analyze events before deteriorations to identify patterns of circulatory and inflammatory response measurements predictive of clinical state deteriorations. These patterns will be compared with those in patients who improve their circulatory status or remain unchanged.

To explore the predictive value of previous measurements to foresee a clinical deterioration, we will use 2 different approaches. The outcome variable will be dichotomized to deterioration versus no deterioration (status quo or improvement). First, we will test different timelags between measurements and deteriorations to see which is more informative (eg, 1 hour, 2 hours, 3 hours). For this analytic approach, we will use univariable and multivariable logistic regression. Second, we will use methods from survival analysis to assess continuous alterations in covariates on the outcome. For this analysis, we will use Aalen’s linear model, which is an intensity regression model. Variables from the 2 groups will also be explored in respect to its relation to mortality and CPC at discharge from hospital.

#### Relation Between Distributive Shock and Inflammatory Biomarkers

We will study the relationship between alterations in inflammatory and endothelial biomarkers and the changes in SVR, in CO, and the need for fluid replacement. As described above, patients with hypotension and low SVR will be treated with norepinephrine. Therefore, we will analyze potential independent biomarkers and other predictors of SVR adjusted for a dose of norepinephrine.

Vasoplegia might also occur as part of an infection, and CA patients are prone to aspiration and pneumonia after CPR. Therefore, we will calculate modified CPIS daily to describe how many patients develop clinical pneumonia.

Circulatory failure might also be due to structural heart defects (eg, mitral valve insufficiency). An experienced cardiologist will perform echocardiogram daily to assess the heart’s function.

## Results

Patient inclusion started in January 2016.

## Discussion

### Rationale for This Study

This study will describe the hemodynamic and inflammatory response characteristics of circulatory failure in PCAS. On the basis of the findings, we will develop a prediction model for risk of circulatory failure in PCAS.

PACS after CA is frequent, and it is strongly associated with mortality after CA [4]. However, there is relatively limited information about the detailed circulatory disturbances in PACS, and principles for circulatory supports are partly transferred from other conditions, in particular, in the treatment of septic shock. Moreover, the expected course (eg, improvement or deterioration) of the circulation is not well understood. The clinical trajectory may be 2-phased: first, a low CO state, followed by a low peripheral resistance state, as previously proposed [20]. Alternatively, it may be one-phased: predominantly caused by either an isolated cardiac failure or an isolated vasoplegia. It is also not established which patients are at a higher risk for circulatory failure in the acute phase of PCAS.

Standard critical illness classification systems, such as SAPS II, SAPS III, acute physiology and chronic health evaluation (APACHE) II, SOFA, or New Early Warning (NEW) scores, are not applicable to describe circulatory changes during the acute phase of PCAS. SAPS and APACHE scores are developed to define risk at admission. SOFA and NEWS scores will reach a ceiling effect and not be able to differentiate between various degrees of circulatory failure in this population. Some researchers have used an extended SOFA circulatory score where 4 further increments are added to the standard circulatory SOFA score [39]. However, this score only includes arterial blood pressure and use of vasopressors, which may not encompass all relevant observations for circulatory stability. Therefore, this study classifies the patients into 3 circulatory groups: *undisturbed, disturbed*, or *severely disturbed circulation* based upon predefined values of mean blood pressure, heart rate, serum lactate concentration, ScvO_2_, use of fluid, vasoactive drugs, and the need for mechanical circulatory devices. Cut-off values were based upon relevant guidelines and clinical practice. All variables were selected because of their known relevance to circulatory failure and because they are easily available during routine monitoring of critically ill patients. More precise measures such as CO or SvO_2_ were not included, as these are usually not obtained in patients after CA.

The clinical trajectories in the acute phase of PCAS are heterogeneous, as seen from the lack of circulatory stability. Such changes can occur immediately after admittance to the ICU or later, at any time, in the clinical course. Thus, the patients are at constant risk. Limited information exists regarding the factors to identify which patients are at the highest risk of imminent clinical circulatory failure. Such factors may include characteristics of the CA episode or clinical observations in the ICU.

This study will establish which factors—demographic, CA-related variables, or clinical observations— will predict circulatory failure and, thus, assess circulatory stability during PCAS.

### Expected Limitations

We recognize some limitations in this protocol. First, the study will obtain information about long-term outcomes and survival. However, this information will only be used to describe the cohort because of the limited number of patients. It would be of interest to study whether circulatory stability and inflammation during PCAS can also predict long-term outcomes, but such analyses should be done in larger cohorts. Second, this study is a single-center study, which limits the generalization of the findings. Third, the included patients are expected to have considerable variability in demographics and comorbidities, in characteristics of the CA, and thus also in PACS complications (eg, infections). Nevertheless, this is presumably the case in all PACS cohorts, and potential findings must be robust to such confounders to be of potential clinical use.

### Conclusions

This study will obtain longitudinally advanced hemodynamic observations with high resolution during the acute phase of PCAS, and it will analyze the details of clinical transitions related to circulatory failure. Additionally, this study will also examine the relationship between inflammatory and endothelial biomarkers and circulatory failure in PCAS.

## Abbreviations

APACHE: acute physiology and chronic health evaluation
CA: cardiac arrest
CCU: coronary care unit
CO: cardiac output
CPC: cerebral performance category
CPIS: clinical pulmonary infection score
CPR: cardiopulmonary resuscitation
EMS: emergency medical service
FiO2: fraction of inspired oxygen
ICU: intensive care unit
IL: interleukin
MAAS: Motor Activity Assessment Scale
MAP: arterial blood pressure
NEW: new early warning
OHCA: out-of-hospital cardiac arrest
PAC: pulmonary artery catheter
PCAS: post cardiac arrest syndrome
PEEP: positive end-expiratory pressure
ROSC: return of spontaneous circulation
SAPS: simplified acute physiology score
SBT: spontaneous breathing trials
SOFA: sequential organ failure assessment
SVR: systemic vascular resistance
TBCS: transitions between circulatory states
